# Cross-species AI: shifting a convolutional neural network from pigs to lambs to detect pneumonia at slaughter

**DOI:** 10.3389/fvets.2025.1591032

**Published:** 2025-05-21

**Authors:** Anastasia Romano, Antonio De Camillis, Domenico Sciota, Simona Baghini, Andrea Di Provvido, Alfonso Rosamilia, Andrea Capobianco Dondona, Nicola Bernabò, Francesca Vaccarelli, Attilio Corradi, Giuseppe Marruchella

**Affiliations:** ^1^Department of Veterinary Medicine, University of Teramo, Teramo, Italy; ^2^Local Health Unit Authority, Teramo, Italy; ^3^Istituto Zooprofilattico Sperimentale della Lombardia e dell’Emilia-Romagna “Bruno Ubertini”, Brescia, Italy; ^4^Farm4trade s.r.l., Atessa, Italy; ^5^Department of Bioscience and Agro-Food and Environmental Technology, University of Teramo, Teramo, Italy; ^6^Department of Communication Science, University of Teramo, Teramo, Italy; ^7^Department of Veterinary Medicine Sciences, University of Parma, Parma, Italy

**Keywords:** pneumonia, pig, lamb, abattoir, postmortem inspection

## Abstract

Abattoir-based data are widely regarded as suitable tools to estimate farm animals’ health and welfare during the entire lifecycles. However, the systematic detection and recording of lesions at postmortem inspection are expensive, time consuming, somewhat biased by inter- and/or intra-observers’ variability. Artificial intelligence could solve the above issues, and it could be particularly well-suited for solving repetitive tasks, by automating workflows and improving their efficiency. This study aims to assess whether a CNN, previously trained to score pneumonia in slaughtered pigs, is likewise capable of solving this task in a different animal species (i.e., in lambs). A total of 229 lamb lungs were photographed at postmortem inspection under different field conditions. Picture were evaluated by 5 independent veterinarians with different professional background, who scored each lung as healthy or diseased. The same pictures were scored by the CNN, which highlighted the lung profile, the bent over lobe (if any), and the lesion (if any). Finally, all veterinarians critically rated CNN’s assessments. Overall, the CNN was able to solve that task, showing a substantial agreement (Cohen’s kappa coefficient between 0.65–0.71) and high level of sensitivity (0.87–0.88), specificity (0.88–0.91), and accuracy (0.87–0.88) when compared to skilled investigators. Shifting CNN to different animal species could facilitate and fasten the adoption of such tools, which could benefit veterinarians and auxiliary staff, mainly where sheep farming is more widespread and economically relevant.

## 1 Introduction

Chronic bronchopneumonia (synonyms “atypical” or “chronic non-progressive” pneumonia) frequently occurs in lambs up to 12-month-old, it is often subclinical and caused by multiple infectious agents (e.g., *Mycoplasma ovipneumoniae* and *Mannheimia haemolytica*), which physiologically reside within the upper respiratory tract. Therefore, its clinical onset and outcome are strongly influenced by a wide range of predisposing factors, including environmental conditions (e.g., sudden weather changes) and lambs’ immune status (1–3). Costs associated with chronic bronchopneumonia can be relevant and mainly result from impaired weight gain, veterinary fees and slaughterhouse waste (4). According to Goodwin-Ray et al. (5), the combined economic losses due to pneumonia and pleurisy averaged NZ$53.2 million in New Zealand in 2003–2004 (i.e., NZ$ 1.36–3.31/lamb).

Chronic bronchopneumonia is considered among the most common pathological findings in slaughtered lambs, even though few data are currently available about its prevalence in European countries. Pleuritis and pneumonia have been reported in 2.8% of slaughtered lambs in Sweden (6), while much higher prevalences have been recorded elsewhere; for instance, on average 28% of lambs showed pneumonic lesions at slaughter in New Zealand (7). At postmortem inspection, lesions affect the cranial lung lobes, and they appear as well demarcated foci of consolidation, red-to-brown in color, with occasional pleuritis (1, 8).

Abattoir-based data are widely recognized as suitable tools to estimate farmed animal health and welfare during the entire lifecycles, and they are often recorded in pigs (9). On the other hand, the systematic detection and recording of lesions at postmortem inspection is expensive, time consuming, somewhat biased by inter- and/or intra-observers’ variability (10, 11).

Artificial intelligence (AI) is currently regarded as a powerful technology in medical sciences. Specifically, convolutional neural networks (CNNs) can analyze complex images to make reliable predictions in digital pathology and diagnostic imaging. AI-based tools are particularly well-suited for solving repetitive tasks, by automating workflows and improving their efficiency. As far as slaughtered animals are concerned, computer vision systems have been developed for meat safety assurance, to detect lesions and carcass contamination in poultry, pigs and cattle (11). To our knowledge, few CNNs have been already developed to score pneumonia (12, 13) and pleurisy in slaughtered pigs (14), while no CNN has been trained to detect and score pneumonia in small ruminants.

The *ad hoc* training of CNNs is a demanding commitment, which requires a lot of time and human resources to build an adequate and well-balanced dataset. This is even more challenging in veterinary medicine, as distinctive morphological features and slaughtering techniques might prevent the application of the same CNN in different animal species. The present study aims to assess whether a CNN, previously trained to score pneumonia in slaughtered pigs, is likewise capable of solving this task in lambs.

## 2 Materials and methods

### 2.1 Deep learning-based method

A previously trained CNN was employed herein, based on a deep learning model using an auto-encoder architecture inspired by U-Net (12). Briefly, U-Net consists of two main parts: (a) the encoder compresses the input image into a smaller, more meaningful representation; (b) the decoder takes the compressed data and expands it back into a full-size image, highlighting the areas of interest. The encoder was based on the ResNet34 model (i.e., Residual Network with 34 layers, designed for image classification) and pre-trained using established classification datasets. The decoder used skip connections to retain more detailed information and learn faster with less data (12, 15).

The CNN was trained with 7,154 pictures of porcine lungs (so called “training set”). Images were taken along the slaughter chain and then evaluated by veterinarians, who annotated three classes using an open-source image segmentation tool (labelme, available at https://github.com/wkentaro/labelme): lung (i.e., the entire silhouette of the lung surface); lesion (i.e., pneumonic foci); lobe (i.e., the cranial lobe when it was bent over, thus partially overlapping with the middle and/or the diaphragmatic lobes). The severity of pneumonia was expressed as a percentage of the entire lung surface:


$$r=\frac{\#\{\text{lesion}\}}{\#\{\text{lung}\}+\#\{\text{lobe}\}}$$

where #{lung}, #{lesion} and #{lobe} represent the number of pixels of each respective class.

The CNN’s performance was assessed in terms of specificity (i.e., the ability to correctly identify healthy lungs), sensitivity (i.e., the ability to correctly identify diseased lungs), and Intersection over Union (IoU), when compared with veterinarians. The IoU is a metric used to measure the accuracy of object detection or image segmentation:


$$\mathrm{IoU}=\frac{\text{Area of Overlap}*}{\text{Area of Union}**}$$

* Common area between the CNN’s prediction and veterinarians’ annotation (i.e., the ground truth region); **Total area covered by both the CNN’s prediction and the ground truth region.

When tested on 410 porcine lung pictures (so called “test set”), CNN’s specificity was 99.38%, sensitivity ranged between 81.25% (lesion size <2% of the entire lung surface) and 100% (lesion size >2% of the entire lung surface), average IoU was around 80% for lesion class (12).

The CNN was not upgraded or fine-tuned to evaluate chronic bronchopneumonia in slaughtered lambs.

### 2.2 Photo collection

**Figure 1:**
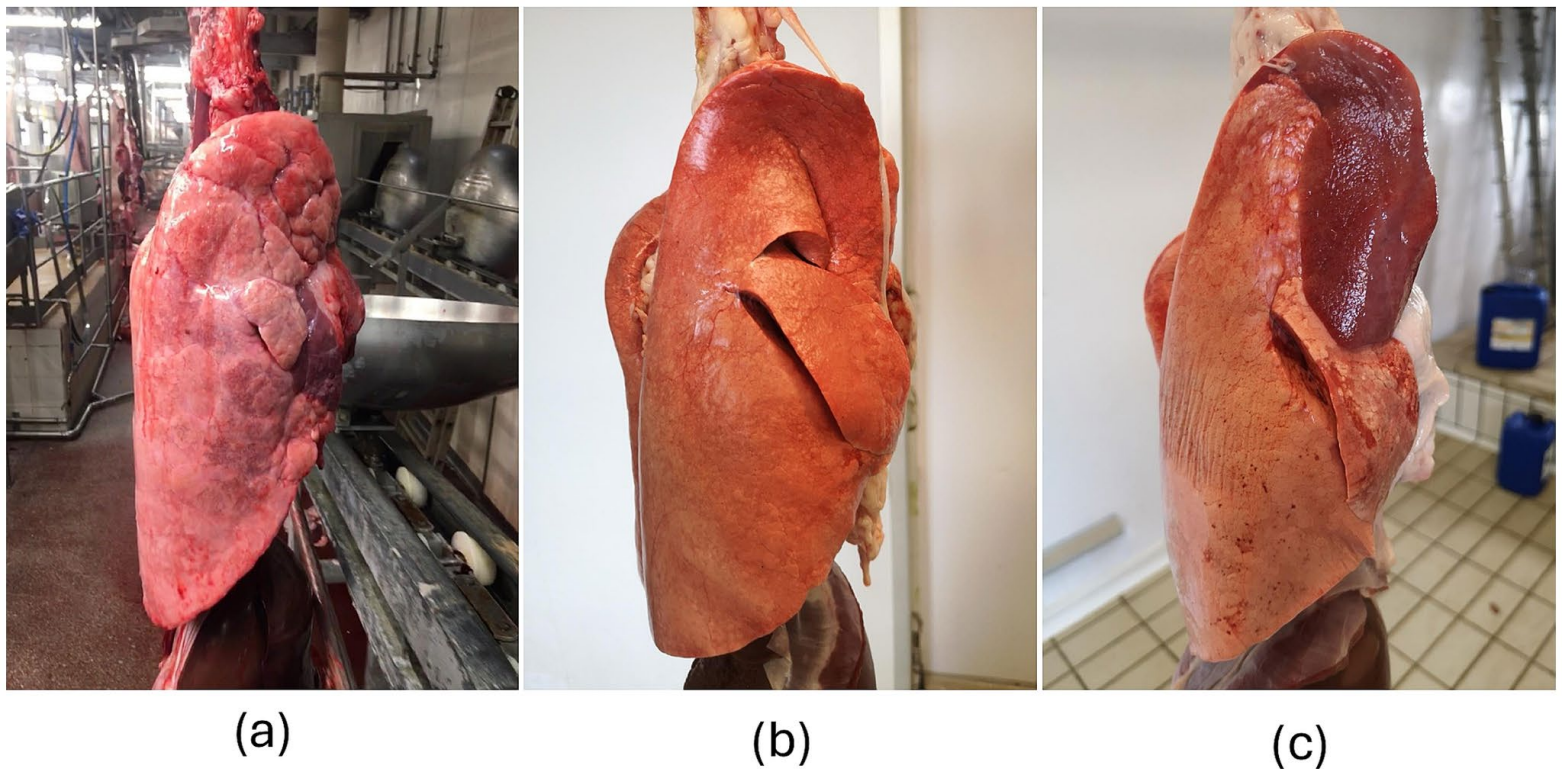
Lungs. (a) Porcine right lung with pneumonia affecting the middle lobe; (b) Healthy right lung of a lamb; (c) Large lesion (bronchopneumonia) affecting the cranial lobe of the lamb’s right lung. The images of lamb lungs share key features with those of porcine lungs, which were previously used to train the CNN (12), providing an optimal view of the lateral surface. However, the background differs significantly, and it could act as a challenging factor. In addition, the appearance of the pig lung (i.e., texture, color) is rather different from that of lambs, owing to morphological features and artifacts due to the slaughtering process.

A total of 229 lamb lungs were photographed at postmortem inspection (see Figure 1 for details), between September and December 2024. Pictures were taken by two veterinarians, in different abattoirs located in the Central and Northern Italy, under different field conditions (e.g., lighting and background).

### 2.3 Image assessment

All pictures were numbered and evaluated by 5 independent veterinarians (namely, A, B, C, D and E), who scored each lung as follows: 0 (healthy), 1 (diseased, i.e., showing chronic bronchopneumonia, regardless of its severity). Veterinarians involved herein have different experience and professional background: A and B have been working for decades in the field of farm animals’ pathology; C is a PhD student studying farm animals’ respiratory disease; D has been working as a swine practitioner for 5 years; E recently graduated in Veterinary Medicine. Notably, veterinarian A contributed to the CNN training (12).

The same pictures were assessed by the CNN, which highlighted the lung profile, the bent over lobe (if any), and the lesion (if any).

**Figure 2:**
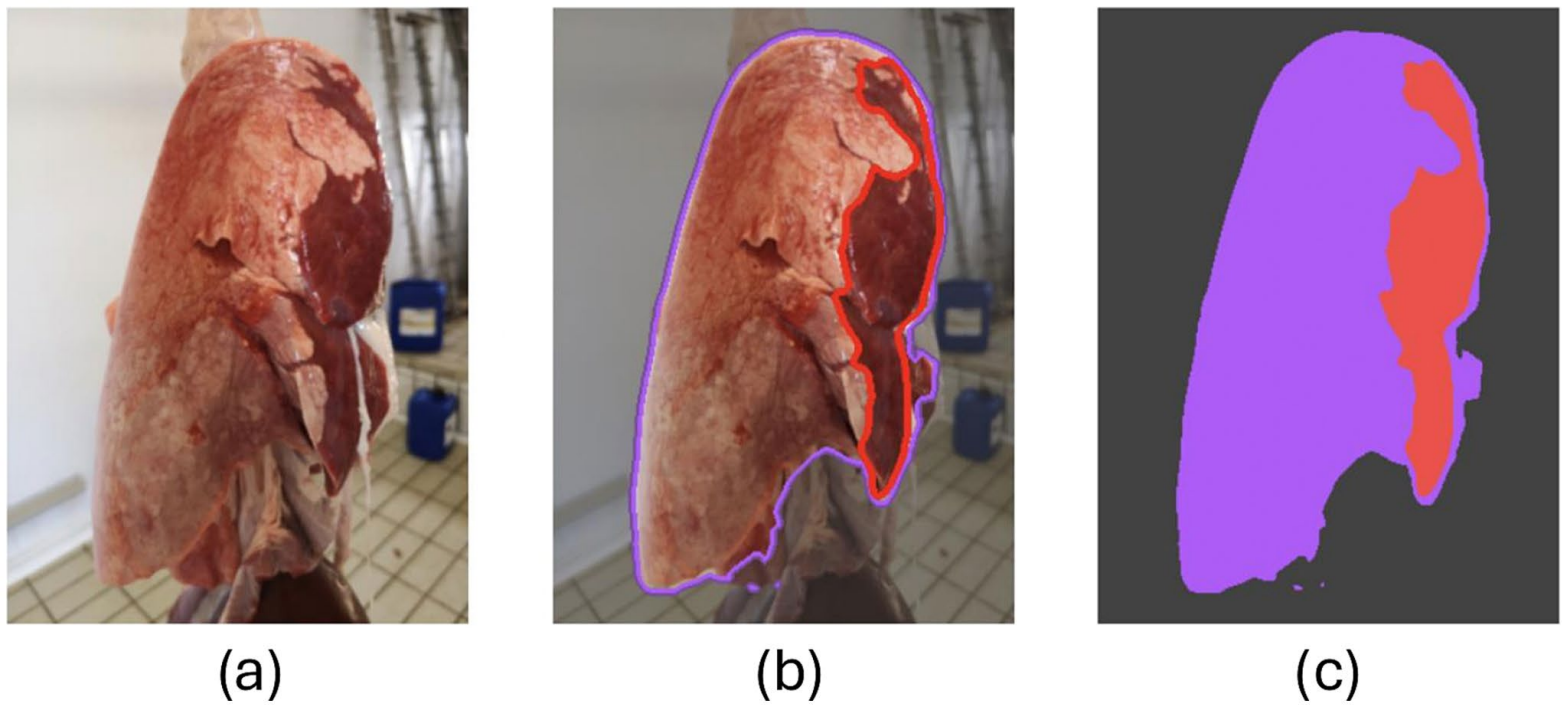
Screenshot of the CNN’s prediction. **(A)** Original picture (input); **(B)** the CNN outlined the lung silhouette (purple line) and a lesion (red line); **(C)** the lung and the lesion are colored in purple and red, respectively.

Moreover, all veterinarians critically evaluated the CNN’s performance (see Figure 2 for details), rating each CNN’s prediction as follows:


$$-0=\text{completely wrong}\;(\begin{array}{l} \text{the}\;\mathrm{CNN}\;\text{was not able to}\;\\ \text{properly identify the lung} \end{array}).$$

$$-1=\text{insufficient}\;(\begin{array}{l} \text{the}\;\mathrm{CNN}\;\text{was not able to detect}\;a\;\text{very evident}\;\\ \text{lesion or misinterpreted}\;a\;\text{healthy lung} \end{array}).$$

$$-2=\text{sufficient}\;(\begin{array}{l} \text{the}\;\mathrm{CNN}\;\text{detected}<50\%\text{of the area}\;\\ \text{of bronchopneumonia} \end{array}).$$

$$-3=\text{good}\;(\begin{array}{l} \text{the}\;\mathrm{CNN}\;\text{detected}\;50–75\%\text{of the area}\;\\ \text{of bronchopneumonia} \end{array}).$$

$$-4=\text{excellent}\;(\begin{array}{l} \text{the}\;\mathrm{CNN}\;\text{correctly identified the}\;\\ \text{healthy lung and}/\text{or the lesion} \end{array}).$$

As a matter of fact, the investigators assessed whether and to what extent the CNN’s prediction overlapped with their hypothetical annotation, with their grades replacing the IoU metric.

### 2.4 Statistical analysis

The CNN’s performance was evaluated in terms of sensitivity [i.e., (true positive)/(true positive + false negative)], specificity [i.e., (true negative)/(true negative + false positive)], accuracy [i.e., (true positive + true negative)/(total number of cases)] and agreement (Cohen’s kappa coefficient), when compared with each veterinarian. The agreement among veterinarians was also assessed (Cohen’s kappa coefficient). In addition, data provided by the CNN (i.e., specificity, sensitivity and accuracy), on one side, and veterinarians, on the other, were analyzed through the Principal Component Analysis.

## 3 Results

Photo assessments provided by veterinarians are summarized in Table 1, while their mutual agreement (Cohen’s kappa coefficient) is reported in Table 2. In addition, explanatory images of healthy and diseased lungs are shown in Figure 1. Overall, the data highlight a sharp divergence between veterinarians A, B, and C on one side, and veterinarians D and E on the other. More specifically, veterinarians A, B, and C classified nearly identical numbers of healthy and diseased lungs, with almost perfect agreement among them (Cohen’s kappa coefficient >0.80) (16). Veterinarians D and E classified a higher number of lungs as diseased and showed fair to moderate agreement with each other and compared to the other investigators (Cohen’s kappa coefficient ranging between 0.38 and 0.60) (16).

**Table 1:** Assessments provided by veterinarians and the CNN on pictures.

	A	B	C	D	E	CNN
Healthy lungs	184	184	185	134	125	168
Bronchopneumonia	45	45	44	95	104	61

Based on the veterinarians’ evaluations, a dichotomous scenario emerges, with investigators A, B, and C providing very similar results. Within this framework, the CNN occupies an intermediate position, although its predictions appear more closely aligned with those of veterinarians A, B, and C.

**Table 2:** Agreement between veterinarians and CNN, computed as pairs.

	A	B	C	D	E	CNN
A		0.91	0.84	0.43	0.38	0.68
B			0.81	0.43	0.39	0.71
C				0.48	0.40	0.65
D					0.60	0.41
E						0.33

Data show an almost perfect agreement between veterinarians A, B, and C (Cohen’s kappa coefficient ranging between 0.81 and 0.91), while it is fair-to-moderate compared to veterinarians D and E. A substantial agreement of the CNN vs veterinarians A, B, and C is observed (Cohen’s kappa coefficient ranging between 0.65 and 0.71), while it is fair-to-moderate vs veterinarians D and E (16).

The CNN classified 168 healthy lungs and 61 diseased lungs. Considering the severity of lesions, computed by the CNN as a percentage of the entire lung surface, it ranged between 0.04 and 33.7% (median value = 5.18%; 25^th^ percentile 1.18%, 75th percentile 14.02%). The CNN interpreted 11 images differently from the unanimous judgment of the veterinarians. More in detail, the CNN identified lesions in 9 lungs, which were interpreted as healthy by all the veterinarians. Conversely, the CNN identified healthy two lungs, which were assessed as diseased by all the veterinarians. Most of such discrepancies resulted from the presence of slaughtering artifacts and/or from shadowed areas on the lung.

Considering CNN’s performance when compared to each veterinarian, data are summarized in Table 2 (Cohen’s kappa coefficient) and Table 3 (sensitivity, specificity, and accuracy). The CNN showed substantial agreement with veterinarians A, B, and C, while agreement was fair with D and E. The CNN’s sensitivity did not vary across veterinarians, whereas its specificity and accuracy were higher when compared to veterinarians A, B, and C than to D and E.

**Table 3:** CNN’s sensitivity, specificity and accuracy, when compared with each single veterinarian.

	A	B	C	D	E
Specificity	0.88	0.91	0.88	0.50	0.45
Sensitivity	0.87	0.88	0.87	0.88	0.87
Accuracy	0.87	0.88	0.87	0.72	0.68

All parameters were expressed as ratio, their value ranging between 0 and 1. Even in this table, a dichotomous scenario is evident. Overall, CNN’s values are very high vs veterinarians A, B, and C, while sensitivity and accuracy values are much lower vs veterinarians D and E.

**Figure 3:**
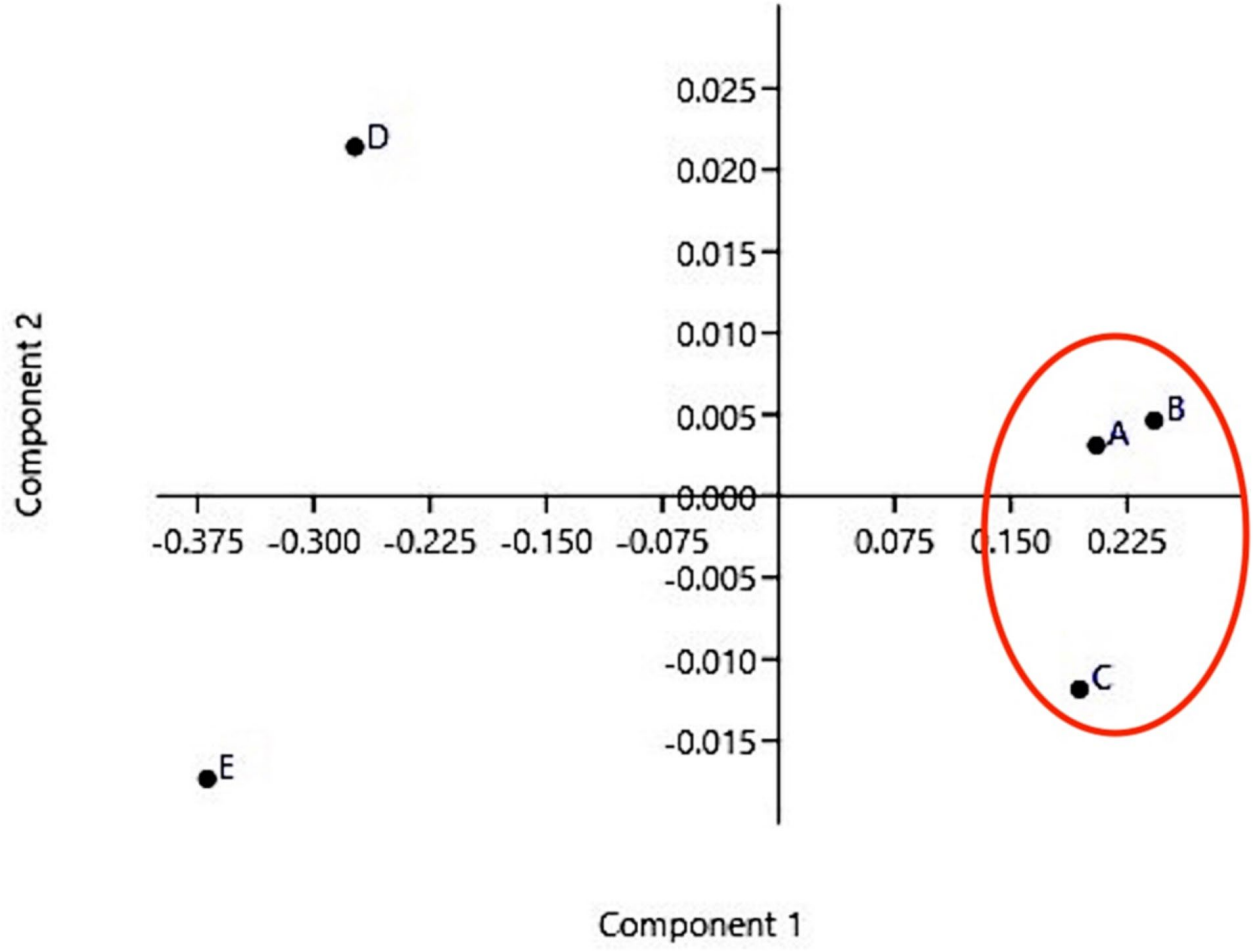
Principal Component Analysis Veterinarians A, B, and C share similar features and cluster together, whereas veterinarians D and E are far from this cluster and from each other.

Principal Component Analysis plot (Figure 3) showed that veterinarians A, B, and C cluster together, thus indicating higher consistency in their assessments.

**Figure 4:**
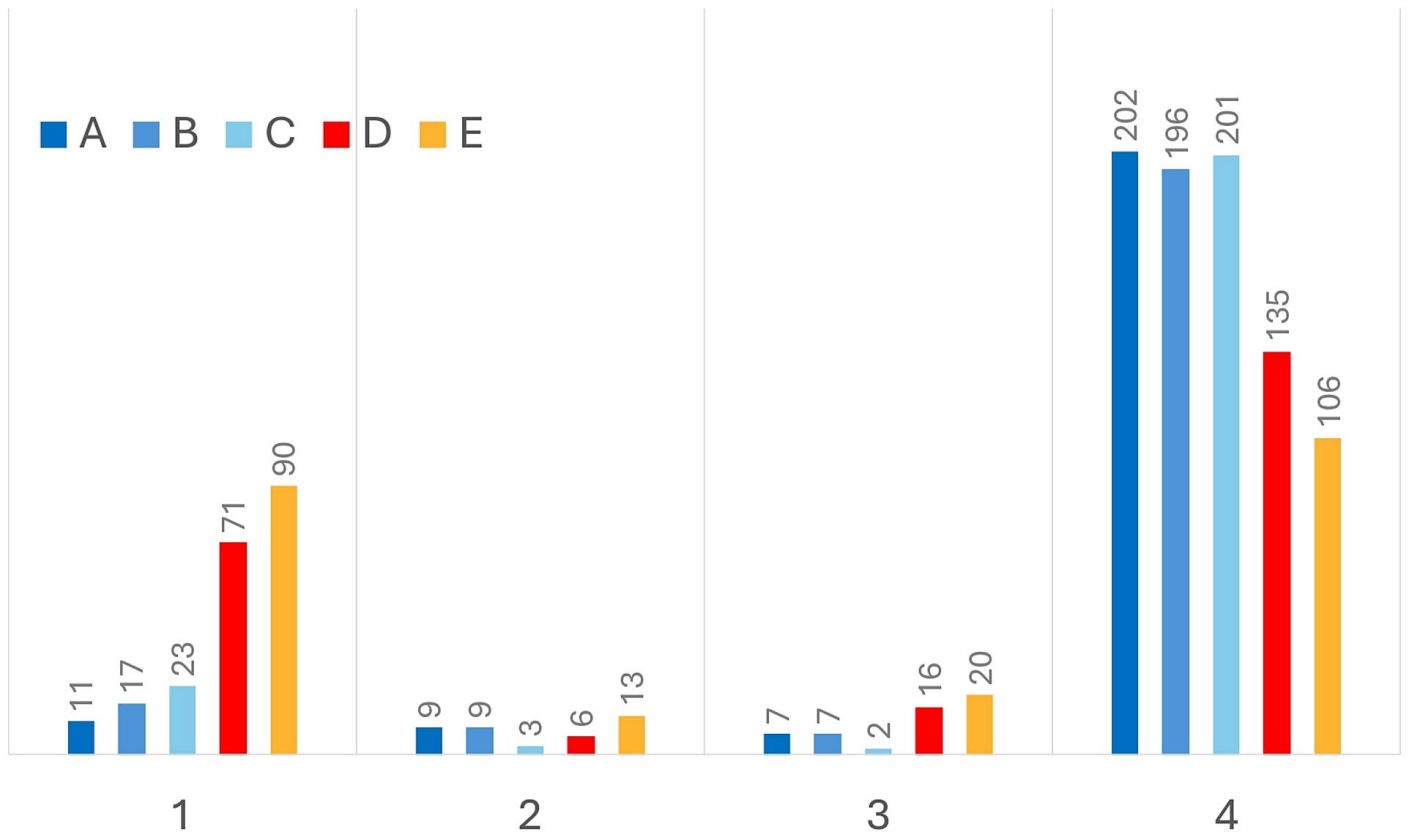
Grades provided by veterinarians. Grade 0 was never assigned, as the CNN was always able to reliably recognize the lung silhouette. Even from this graphic a dichotomous scenario emerges. Veterinarians A, B, and C assigned the highest score (i.e., grade 4) to 85.5–88.2% of CNN predictions, while investigators C and D more frequently considered insufficient the CNN performance (i.e., grade 1).

Finally, the grades assigned by veterinarians to the CNN are presented graphically in Figure 4 (maximum possible score = 916). Veterinarians A, B, and C gave the highest ratings to the CNN, with overall scores of 858, 840, and 839, respectively. Veterinarians D and E rated the CNN’s performance less favorably, with overall scores of 671 and 600, respectively.

## 4 Discussion

Artificial intelligence is shaping most human activities, including biomedical sciences. In this respect, deep learning models are emerging as suitable tools, which could support clinicians, pathologists and official veterinarians in the framework of their diagnostic approach (e.g., at postmortem inspection). In this case, AI-based tools could be particularly helpful for performing repetitive tasks, where thousands of animals are slaughtered at high processing line speeds (11, 17).

During the last few decades, several AI-based methods have been developed to identify and score lesions in slaughtered animals, useful to approve meat for human consumption and/or as welfare and health indicators (11). Training CNNs for these purposes is often a long and challenging process, it needs the collection of large and well-balanced datasets (e.g., thousands of pictures collected under various field conditions), demanding significant efforts to annotate images and to manage inter-raters’ variability (18). This is even more difficult as each animal species has its own anatomic features. For instance, porcine lungs show a more pronounced texture of interlobular connective tissue, when compared to small ruminant lungs (19). Likewise, slaughtering techniques vary significantly (e.g., pigs usually pass through the scalding tank), thus leading to more or less evident artifacts, which can further complicate the detection of lesions and their interpretation. The age of slaughtered animals is an additional critical factor, as it affects the appearance of lesions at postmortem inspection; bronchopneumonia is strongly hyperemic during the acute stage of the disease, while it becomes grayish and shrinks over time (20).

This study aimed to assess the possibility of shifting CNN to different animal species (namely, from pig to lamb), as cross-species transfers could facilitate and fasten the adoption of such tools. Overall, we consider that the CNN employed herein showed satisfactory performance, even though lower when compared to those achieved in slaughtered pigs (12). Worthy of note, the CNN showed a substantial agreement (Cohen’s kappa coefficient between 0.65–0.71) and reached higher levels of sensitivity, specificity, and accuracy when compared to investigators with greater experience in farm animals’ respiratory diseases (A, B, and C), regardless they contributed to its training. Veterinarians D and E overestimated the prevalence of bronchopneumonia, mostly due to the misinterpretation of slaughter artifacts. The same artifacts were responsible of most discrepancies between the CNN, on one side, and all veterinarians, on the other. In our opinion, this implies two relevant considerations. First, it further supports the reliability of this CNN, which ranks among the most “skilled” veterinarians. In addition, it highlights inter-raters’ variability as a serious concern, which can be mitigated (never eliminated) through continuous training and joint sessions. This concern is even more relevant when evaluations only rely on visual inspection of lungs (21). Considering this, the use of AI-based tools could provide an additional advantage, ensuring fully standardized assessments.

In conclusion, this study suggests that shifting a CNN from one animal species to another could be a feasible and advantageous approach, reducing time, efforts and costs for new applications. Additional training with a targeted dataset (i.e., pictures of lamb lungs) would allow to enhance CNN’s performance further. The development of such AI-based technologies could benefit veterinarians and auxiliary staff, especially in regions where sheep farming is more relevant and widespread.

## Data Availability

The original contributions presented in the study are included in the article/supplementary material, further inquiries can be directed to the corresponding author/s.
